# New Appliances and Things Medical

**Published:** 1898-08-06

**Authors:** 


### NEW APPLIANCES AND THINGS MEDICAL.

[We shall be glad to receive, at our Office, 28 & 29, Southampton Street, Strand, London, W.C., from the manufacturers, specimens of all new preparations and appliances which may be brought out from time to time.]

### DIASTASED FARINA.

(Messrs. E. Beanes and Co., Falcon Works, Hackney Wick, E.)

Diastasic food has been found to be very beneficial when used in the feeding of children or in the dietary of invalids suffering from weak digestion. "Diastased Farina" is a well-prepared farinaceous food containing a large proportion of active diastase, in addition to which it contains a satisfactory amount of soluble carbohydrates and albumenoids. The mineral constituents, chiefly phosphates—an important consideration in the dietary of children—amounted to 1·8 per cent. It makes a very palatable food either as porridge or in other ways, and can be recommended with confidence for the use of children and invalids.

### OREXIN.

(A. and M. Zimmerman, 9 and 10, St. Mary at-Hill, E.C.)

Orexin, chemically known as phenyldihydrochinazolin, is one of the synthetic remedies which appears to have a claim for a place in the materia medica as a stomachic and appetiser. It is a slightly bitter substance, and can be taken either in its basic form or in the form of its salts, such as the hydrochloride or tannate. Orexin has a marked action on the appetite of a healthy person after the first dose, but with patients the improvement is more manifest after a few days. In many clinical cases described by Professor Penzoldt the appetite and digestion were stimulated, whilst Dr. Bodenstein has had great success when prescribing orexin in the form of tannate in anorexia of phthisis, anæmia, and chlorosis, as well as in cases of atony of the stomach. It is prepared in the form of chocolate tablets, each tablet containing 4 grains of orexin tannate, and in 3-grain pills with 1½ grains of orexin. The dose of the tablets is 2-4, to be taken 1½-2 hours before the mid day and evening meals in a little liquid, continuing the dose for four or five consecutive days. As it is stated that the prolonged use of orexin and its preparations has no deleterious results further trials are certainly worth consideration.

### "AMIRAL" SOAP.

(The Amiral Soap Syndicate, 28a, Basinghall Street, E.C.)

This is a somewhat strongly-scented, green-coloured neutral soap containing certain biliary compounds. It is claimed that its effect is to remove, or, at any rate, lessen the adipose tissue over which it is applied. No doubt the idea is founded on the well-known power possessed by bile of emulsifying fats, but how far in the first instance the soap can be absorbed by the skin, and how far in the process of absorption the biliary compound which it contains would have the opportunity of influencing the subcutaneous fat, are matters concerning which we have no evidence. The probabilities are much against any such action. The soap, however, seems free from any injurious properties, and it is a perfectly fair thing to try.

